# Maintenance of Remission and Risk of Relapse in Myeloperoxidase-Positive ANCA-Associated Vasculitis with Kidney Involvement

**DOI:** 10.2215/CJN.06460622

**Published:** 2022-01-18

**Authors:** Marta Casal Moura, Ulrich Specks, Shahrzad Tehranian, Sanjeev Sethi, Dalia Zubidat, Luca Nardelli, Fernanda G. dos Santos, Ciria Sousa, Juan León-Róman, Shane A. Bobart, Eddie Greene, Ladan Zand, Fernando C. Fervenza

**Affiliations:** 1 Division of Pulmonary and Critical Care, Department of Medicine, Mayo Clinic College of Medicine and Science, Rochester, Minnesota; 2 Departamento de Biomedicina, Faculdade de Medicina da Universidade do Porto, Porto, Portugal; 3 Division of Nephrology and Hypertension, Department of Medicine, Mayo Clinic College of Medicine and Science, Rochester, Minnesota; 4 Division of Anatomic Pathology, Department of Laboratory Medicine and Pathology, Mayo Clinic College of Medicine and Science, Rochester, Minnesota; 5 Department of Nephrology and Hypertension, Cleveland Clinic Florida, Weston, Florida

## Abstract

**Background:**

The optimal strategy for remission-maintenance therapy in patients with myeloperoxidase-ANCA (MPO-ANCA)–associated vasculitis is not established. Defining parameters to guide maintenance therapy is required.

**Methods:**

This was a retrospective cohort study of all patients with MPO-ANCA–associated vasculitis (microscopic with polyangiitis and granulomatosis with polyangiitis) and GN followed at the Mayo Clinic between 1996 and 2015. Relapse rate, MPO-ANCA status, and remission-maintenance therapies were reviewed. Logistic regression models, Kaplan–Meier method, and Cox proportional hazards regression models were applied.

**Results:**

We analyzed 159 patients with active MPO-ANCA–associated vasculitis with GN. Sixty-six (42%) patients had at least one relapse, and 52 (33%) relapsed before 60 months. Patients with MPO-ANCA who became persistently negative did not relapse (hazard ratio [HR], 0.03; 95% confidence interval [95% CI], 0.002 to 0.431; *P*=0.01). The reappearance of MPO-ANCA was associated with a higher risk of relapse (HR, 1.91; 95% CI, 1.109 to 3.293; *P*=0.02). Immunosuppression was withdrawn in 80 (50%) patients, and this was less likely in those who received cyclophosphamide for remission induction or in patients with persistently positive MPO-ANCA (odds ratio [OR], 0.44; 95% CI, 0.228 to 0.861; *P*=0.02 and OR, 0.42; 95% CI, 0.213 to 0.820; *P*=0.01, respectively). Relapse frequency was not different between patients with persistently positive MPO-ANCA and patients with MPO-ANCA reappearance (44% versus 39%, *P*=0.49), irrespective of remission-maintenance treatment. Ear, nose, and throat involvement (OR, 6.10; 95% CI, 1.280 to 29.010; *P*=0.02) and MPO-ANCA reappearance (OR, 9.25; 95% CI, 3.126 to 27.361; *P*<0.001) were independently associated with relapse after treatment withdrawal.

**Conclusions:**

Patients persistently MPO-ANCA negative are at low risk for relapse even without remission-maintenance therapy. Persistence or subsequent reappearance of MPO-ANCA is associated with a higher risk of relapse.

**Podcast:**

This article contains a podcast at https://dts.podtrac.com/redirect.mp3/www.asn-online.org/media/podcast.aspx?p=CJASN&e=2023_01_10_CJN06460622.mp3

## Visual Abstract

**Figure:**
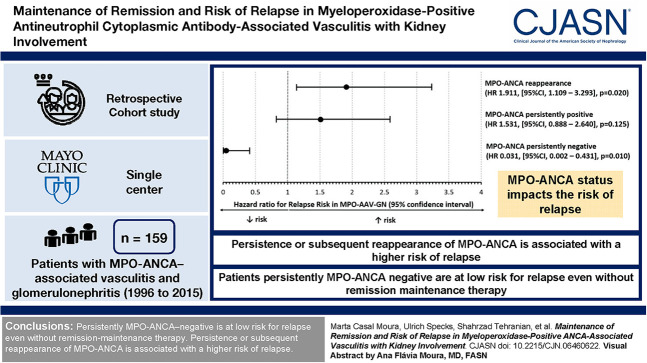

## Introduction

Myeloperoxidase (MPO) ANCAs are commonly associated with predominantly vasculitic features and kidney involvement in patients with ANCA-associated vasculitis (AAV).^1^ Advances in remission-induction treatment regimens resulted in significant survival improvements.^2–5^ However, it is important to determine which patients need maintenance immunosuppression, the optimal duration of therapy, and whether withdrawal of remission maintenance is possible without increasing the relapse risk.^6–8^

The main goals of remission-maintenance treatment are prevention of relapse while avoiding long-term drug toxicities and comorbidities.^9^ The decision to withdraw remission-maintenance treatment is complicated by the potential risk of relapse and the absence of specific guidance criteria.^6–8,10^ Relapse is expected to occur in 30%–50% of the patients with ANCA-associated vasculitis, and GN may result in progressive kidney function loss and reduced kidney survival.^3,5^ Several studies have shown that PR3-ANCA conveys a higher relapse risk than MPO-ANCA.^2,11–17^ In the most recent guidelines on ANCA-associated vasculitis management, recommendations about the monitoring of ANCA levels are based on conclusions of a meta-analysis that included studies in which the relapse risk was assessed in cohorts that combined patients with MPO- and PR3-AAV.^8,18^ Importantly, the number of relapses in the MPO-ANCA–associated vasculitis subsets of most included studies has been considered too low for conclusions about the utility of MPO-ANCA monitoring for relapse prediction.^8^

However, subsequent studies indicated that MPO-ANCA levels were associated with relapses, particularly in patients with AAV-GN.^15,19–21^ Taken together, serial determinations of ANCAs might be of interest for guiding remission-maintenance treatment strategies in patients with AAV-GN, particularly in MPO-ANCA–associated vasculitis with GN. Consequently, we hypothesized that remission-maintenance treatment strategies could be tailored according to ANCA specificity and ANCA status during follow-up.

To address this hypothesis, we evaluated patients with MPO-ANCA–associated vasculitis with GN and analyzed: (*1*) the remission-maintenance treatments used in routine clinical practice and their outcomes and (*2*) how relapses are related to the MPO-ANCA status.

## Methods

Study design, definitions, outcomes, and methodology are described in detail in the Supplemental Material. Herein, we provide a summary.

### Study Design

A single-center retrospective cohort study of consecutive patients with positive MPO-ANCA–associated vasculitis with active kidney disease evaluated at Mayo Clinic from January 1, 1996, to December 31, 2015.^22^

### Patient Characteristics

The clinicopathologic diagnosis originally assigned by the clinician was accepted. Inclusion required known remission status, defined remission-maintenance strategy, and 6 months of follow-up after achieving remission.

### Outcome Assessment

Remission was defined as Birmingham Vasculitis Activity Score/Wegener Granulomatosis=0 independent of prednisone dose, and sustained remission was defined by Birmingham Vasculitis Activity Score/Wegener Granulomatosis=0 for more than 6 months independently of prednisone dose. Relapse was defined as the increase of Birmingham Vasculitis Activity Score/Wegener Granulomatosis ≥1 after remission was achieved, resulting in reinitiating or change of immunosuppressive therapy. Patients had to have achieved remission to be included in any of the relapse analyses.

### MPO-ANCA Status

Patients were longitudinally analyzed and classified according to MPO-ANCA status at the end of the follow-up: (*1*) persistently negative, (*2*) reappearance of MPO-ANCA, and (*3*) persistently positive.

### Statistical Analysis

Categorical variables were presented as count (percent), normally distributed continuous variables were presented as mean (SD), and not normally distributed variables were presented as median (interquartile range [IQR]). Pearson's chi-square test was used to compare categorical variables between groups; Bonferroni correction was applied when necessary. For the comparison of continuous variables between groups, Student's *t*, Mann–Whitney *U*, ANOVA, and Kruskal–Wallis tests were used depending on the number of compared groups and distribution of variables.

Logistic regression models, the Kaplan–Meier method, and Cox proportional hazards regression models were used as necessary.

IBM SPSS Statistics for MacOS, version 26 (IBM, Armonk, NY), was used for all data analysis.

## Results

### Patient Characteristics and Clinical Outcomes

Of the 1830 patients with ANCA-associated vasculitis evaluated during the study period, 251 patients had MPO-ANCA–associated vasculitis with active kidney involvement. Of these, 92 were excluded because of anti-GBM positivity, early death, <6 months of follow-up, or no MPO-ANCA determinations, and 159 (63%) were included (Supplemental Figure 1). Baseline demographics and outcomes are presented in Supplemental Tables 1 and 2. Most of the patients had newly diagnosed ANCA-associated vasculitis with GN (131, 82%). At diagnosis, mean eGFR was 25 (IQR 15.7–43.3) ml/min per 1.73 m^2^; 37 (23%) patients had an eGFR <15 ml/min per 1.73 m^2^, and 19 (12%) patients required hemodialysis. Cyclophosphamide was the remission-induction immunosuppressant in 80 (50%) patients, followed by rituximab in 43 (27%) patients, mycophenolate mofetil (MMF) in 27 (17%) patients, oral prednisone alone in seven (4%) patients, and methotrexate in two (1%) patients. Plasma exchange was added to remission-induction treatment in 20 (13%) patients. Remission (Birmingham Vasculitis Activity Score/Wegener Granulomatosis=0) was achieved by 6 months in 130 (82%) patients. At the exact time of remission achievement (median 4.1 [IQR 2.7–5.9] months), 46 (29%) patients had become MPO-ANCA–negative and 90 (57%) patients in total became MPO-ANCA–negative at some point during follow-up. The median time to achieve the MPO-ANCA–negative status was 3.0 (IQR 0.5–9.6) months after initiation of remission-induction therapy. By the end of the follow-up (median of 7.7 [IQR 4.4–12.1] years), a total of 44 (28%) patients had died.

### Remission Maintenance

Of the 159 patients with MPO-ANCA–associated vasculitis with GN, MMF was used as a remission-maintenance immunosuppressant in 61 (38%) patients, azathioprine in 53 (33%) patients, low-dose oral prednisone alone in 26 (16%) patients, rituximab in 11 (7%) patients, methotrexate in six (4%) patients, and cyclophosphamide in two (1%) patients (Supplemental Table 1). The median duration of remission-maintenance treatment was 31.2 (IQR 11.8–88.2) months. Remission-maintenance therapy was continued throughout the entire follow-up in 79 (50%) patients. In the remaining 80 (50%) patients, remission-maintenance treatment was withdrawn: 64 (80%) patients were in sustained remission; five (6%) patients had developed an adverse reaction; four (5%) patients had other medical conditions requiring discontinuation; and two (3%) patients discontinued the drug by themselves.

### Relapse Characterization

A total of 66 (42%) patients had at least one relapse (index) during follow-up, and 52 (33%) patients relapsed before 60 months. Overall, relapses were major in 57 (36%) patients and with confirmed kidney involvement in 42 (27%) patients. The overall median time to relapse was 38.9 (IQR 12.1–58.8) months (Supplemental Table 2). Patients who relapsed had higher mean eGFR at diagnosis (28 versus 25 ml/min per 1.73 m^2^, *P*=0.02) and lower frequency of patients with kidney failure at diagnosis (14% versus 28%, *P*=0.04) when compared with those who did not relapse (Table 1). Patients with MPO-ANCA reappearance had a higher frequency of relapse (48% versus 28%, *P*=0.01), whereas patients with persistently positive MPO-ANCA were similarly distributed in both groups (52% versus 39%, *P*=0.13) (Table 1). The median eGFR loss among the patients who relapsed was 5 ml/min per 1.73 m^2^, but that did not translate into differences in the frequency of kidney failure or combined events of kidney failure and/or death between groups at follow-up.

**Table 1:** Clinical characteristics of patients with myeloperoxidase-ANCA–associated vasculitis with glomerulonephritis according to the occurrence of relapse over 60 months (*n*=159)

	No Relapse *n*=107 (67%)	Relapse *n*=52 (33%)
Age at diagnosis, median (IQR) yr	66 (5 –74)	59 (53.7–69.4)
Male, *n* (%)	44 (41)	25 (48)
**Presentation, *n* (%)**		
New diagnosis	87 (81)	44 (84.6)
Relapse	20 (19)	8 (15)
**AAV, *n* (%)**		
MPA	96 (90)	48 (92)
GPA	11 (10)	4 (8)
BVAS/WG at diagnosis, median (IQR)	7 (7–9)	8 (7–10)
Alveolar hemorrhage BVAS/WG at diagnosis, *n* (%)	18 (17)	12 (23)
**Cardiovascular risk factors, *n* (%)**		
Arterial hypertension	76 (71)	35 (67)
Diabetes mellitus	14 (13)	7 (14)
Dyslipidemia	41 (38)	17 (33)
BMI >30 kg/m^2^ (obese)	33 (34)	23 (46)
**Laboratory findings**		
Hemoglobin, mean (SD), g/dl	10.6 (1.9)	10.3 (1.8)
eGFR at diagnosis, median (IQR), ml/min per 1.73 m^2^	25 (15–441)	28 (16–42)
eGFR at diagnosis <30 ml/min per 1.73 m^2^, *n* (%)	67 (63)	25 (48)
eGFR at diagnosis <15 ml/min per 1.73 m^2^, *n* (%)	30 (28)	7 (14)
**Remission-induction therapies, *n* (%)**		
Cyclophosphamide	57 (53)	23 (44)
Rituximab	28 (26)	16 (31)
MMF	17 (16)	10 (19)
**Remission-induction adjuvant therapies, *n* (%)**		
IV methylprednisolone at induction remission	62 (58)	28 (54)
Plasma exchange therapy	11 (10)	9 (17)
**Maintenance treatment, *n* (%)**		
MMF	41 (40)	20 (40)
Azathioprine	36 (35)	18 (36)
Prednisone only	18 (18)	8 (16)
Rituximab	8 (8)	3 (6)
Methotrexate	4 (4)	7 (14)
Cyclophosphamide	2 (2)	0 (0)
Time of remission-maintenance therapy, median (IQR)	30.6 (13.2–81.2)	32.0 (12.2–121.7)
**ANCA seronegative conversion after remission induction, *n* (%)**		
At remission	35 (32.7)	11 (21.2)
Total	65 (60.7)	25 (48.1)
Time to turn MPO-ANCA–negative (IQR), mo	2.0 (0.0–6.0)	1.4 (0.0–15.2)
**ANCA profile over time, *n* (%)**		
Persistently negative	35 (33)	0 (0)
Reappearance	30 (28)	25 (48)
Persistently positive	42 (39)	27 (52)

IQR, interquartile range; AAV, ANCA-associated vasculitis; MPA, microscopic polyangiitis; GPA, granulomatosis with polyangiitis; BVAS/WG, Birmingham vasculitis activity score for Wegener granulomatosis; BMI, body mass index; MMF, mycophenolate mofetil; IV, intravenous; MPO, myeloperoxidase.

Using the Kaplan–Meier method, we found no difference in the time to relapse or kidney relapse up to 60 months when comparing the three main remission-maintenance agents used (Supplemental Figure 2) and the different remission-induction strategies (Supplemental Figure 3).

**Figure 1:**
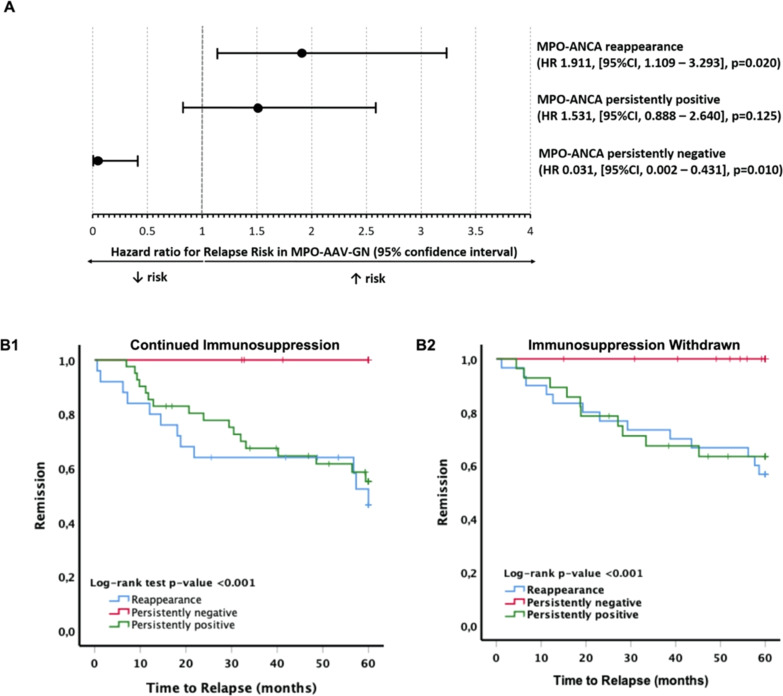
(A) Predictive value of MPO-ANCA status for relapse at 60 months: MPO-ANCA reappearance after turning negative is associated with a higher risk for relapse, whereas persistent MPO-ANCA positivity was not, and persistently negative MPO-ANCA status was negatively associated with relapse (hazard ratio [HR], 0.028; 95% confidence interval [95% CI], 0.003 to 0.272; *P*=0.002). (B) Kaplan–Meier plots of relapse over 60 months according to MPO-ANCA status. (B1) In patients who were maintained under remission maintenance. (B2) In patients who stopped remission maintenance. MPO, myeloperoxidase.

By unadjusted Cox regression, we determined that, compared with patients who did not relapse over 60 months (*n*=107), higher body mass index (hazard ratio [HR], 1.07; 95% confidence interval [95% CI], 1.016 to 1.127; *P*=0.01), presence of lung infiltrates at the time of diagnosis (HR, 2.09; 95% CI, 1.175 to 3.699; *P*=0.02), eGFR at diagnosis (HR, 1.01; 95% CI, 1.001 to 1.022; *P*=0.03), eGFR at 12 months (HR, 1.10; 95% CI, 1.002 to 1.022; *P*=0.02), and MPO-ANCA reappearance after turning negative (HR, 1.91; 95% CI, 1.109 to 3.293; *P*=0.02) were predictive factors of relapse over 60 months. Persistent MPO-ANCA positivity was not a predictive factor for relapse (HR, 1.53; 95% CI, 0.888 to 2.640; *P*=0.12). Persistently negative MPO-ANCA was contrariwise associated with relapse (HR, 0.03; 95% CI, 0.002 to 0.431; *P*=0.01). Using multivariable Cox regression, we determined that MPO-ANCA reappearance; presence of ear, nose, and throat (ENT) involvement; body mass index; and eGFR at 12 months were independently associated with relapses at 60 months (Figure 1A and Table 2).

**Table 2:** Multivariable analysis of predictive factors for relapse at 60 months in patients with myeloperoxidase-ANCA–associated vasculitis with glomerulonephritis

	Multivariable Cox Regression	
	HR (95% CI)	*P Value
**Relapses**		
MPO-ANCA reappearance	2.82 (1.411 to 5.648)	0.01
ENT involvement	2.24 (1.026 to 4.877)	0.04
BMI (kg/m^2^)	1.09 (1.022 to 1.164)	0.01
eGFR at 12 mo (ml/kg per 1.73 m^2^)	1.02 (1.004 to 1.028)	0.01
Kept remission-maintenance treatment	1.77 (0.870 to 3.582)	0.12

HR, hazard ratio; 95% CI, 95% confidence interval; MPO, myeloperoxidase; ENT, ear-nose-throat; BMI, body mass index. *P value <0.05 is considered significant (multivariable Cox regression).

### MPO-ANCA Status Classification after Remission-Induction Therapy

Patients were classified according to their MPO-ANCA status as follows: (*1*) patients who turned and remained MPO-ANCA–negative, 35 (22%); (*2*) patients who turned MPO-ANCA–negative but subsequently turned positive again, 55 (35%); and (*3*) patients who remained MPO-ANCA–positive during follow-up, 69 (43%) (Table 3). There were no differences in the clinical presentation or demographics, except for the number of patients with body mass index >30 kg/m^2^, which was significantly more frequent among patients who remained MPO-ANCA–positive throughout the follow-up (*P*=0.01). Of the 90 patients who turned MPO-ANCA–negative, 55 (61%) patients exhibited MPO-ANCA reappearance (median time, 5.5 [IQR 3.1–15.4] months).

**Table 3:** Clinical characteristics of patients with myeloperoxidase-ANCA–associated vasculitis with glomerulonephritis according to myeloperoxidase-ANCA status (*n*=159)

	Persistent MPO-ANCA–Negative *n*=35 (22.0%)	MPO-ANCA Reappearance *n*=55 (34.6%)	Persistent MPO-ANCA–Positive *n*=69 (43.4%)	*P* Value^a^
Age at diagnosis, median (IQR), yr	67 (59–78)	64 (50–71)	65 (56–73)	0.26
Male, *n* (%)	12 (34)	24 (44)	33 (48)	0.42
**Presentation, *n* (%)**				0.41
New diagnosis	31 (89)	46 (84)	54 (78)	
Relapse	4 (11)	9 (16)	15 (22)	
**AAV, *n* (%)**				0.90
MPA	32 (91)	49 (89)	63 (91)	
GPA	3 (9)	6 (11)	6 (8.7)	
BVAS/WG at diagnosis, median (IQR)	7 (7–10)	7 (6–9)	7 (7–9)	0.66
Alveolar hemorrhage BVAS/WG at diagnosis, *n* (%)	6 (17)	10 (18)	14 (20)	0.92
**Cardiovascular risk factors, *n* (%)**				
Arterial hypertension	27 (77)	33 (60)	51 (74)	0.14
Diabetes mellitus	3 (9)	4 (7)	14 (20)	0.07
Dyslipidemia	12 (34)	19 (35)	27 (39)	0.83
BMI >30 kg/m^2^	9 (27)	14 (29)	33 (52)	0.01
**Laboratory findings**				
Hemoglobin, mean (SD), g/dl	10.5 (1.8)	10.3 (1.6)	10.9 (2.1)	0.14
eGFR at diagnosis, median (IQR), ml/min per 1.73 m^2^	20 (11–43)	50 (16–41)	28 (17–43)	0.69
eGFR at diagnosis<30 ml/min per 1.73 m^2^, *n* (%)	22 (63)	32 (58)	38 (55)	0.75
eGFR at diagnosis<15 ml/min per 1.73 m^2^, *n* (%)	10 (29)	13 (24)	14 (20)	0.63
**Remission-induction therapies, *n* (%)**				
Cyclophosphamide	21 (60)	31 (57)	28 (41)	0.10
Rituximab	7 (20)	11 (22)	25 (36)	0.11
MMF	5 (14)	9 (17)	13 (19)	0.83
**Remission-induction adjuvant therapies, *n* (%)**				
IV methylprednisolone at induction remission	19 (54)	29 (53)	42 (61)	0.63
Plasma exchange therapy	4 (11)	4 (7)	12 (17)	0.23
**Maintenance treatment, *n* (%)**				0.04
MMF	13 (37)	20 (36)	28 (41)	
Azathioprine	13 (37)	23 (42)	18 (26)	
Prednisone	7 (20)	8 (15)	10 (15)	
Rituximab	0 (0)	3 (6)	8 (12)	
Methotrexate	0 (0)	1 (2)	5 (7)	
Cyclophosphamide	2 (6)	0 (0)	0 (0)	

MPO, myeloperoxidase; IQR, interquartile range; AAV, ANCA-associated vasculitis; MPA, microscopic polyangiitis; GPA, granulomatosis with polyangiitis; BVAS/WG, Birmingham vasculitis activity score for Wegener granulomatosis; BMI, body mass index; MMF, mycophenolate mofetil; IV, intravenous.

a *P* value <0.05 is considered significant (Pearson χ^2^ test for categorical variables, ANOVA test for continuous variables normally distributed, and Kruskal–Wallis test for continuous variables with skewed distribution).

**Figure 2:**
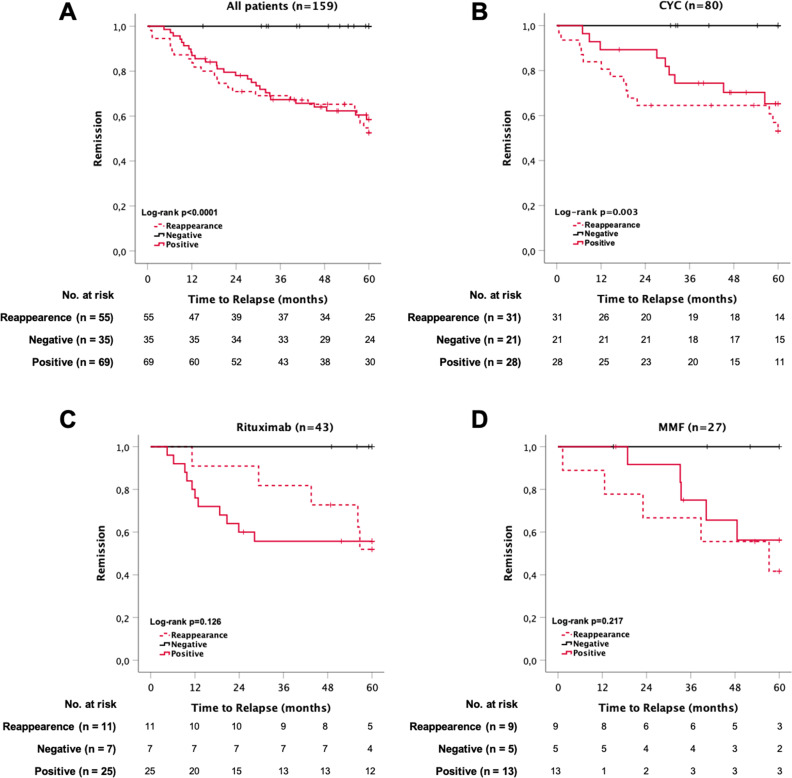
**Kaplan**–**Meier plots of relapse over 60 months according to their MPO-ANCA status after remission-induction treatment (MPO-ANCA reappearance versus persistent MPO-ANCA seronegative versus persistent MPO-ANCA–positive).** (A) All patients—25 versus 0 versus 27 events, mean time to event—23 versus 0 versus 13 months, *P*<0.001. (B) According to remission-induction treatment with cyclophosphamide—14 versus 0 versus 9 events, mean time to event—19 versus 0 versus 13 months, *P*=0.003. (C) According to remission-induction treatment with rituximab—5 versus 0 versus 11 events, mean time to event—34 versus 0 versus 9 months, *P*=0.126. (D) According to remission-induction treatment with MMF—5 versus 0 versus 5 events, mean time to event—28 versus 0 versus 18 months, *P*=0.22.

Using the Kaplan–Meier method, we determined that MPO-ANCA status was related to the incidence of relapse over 60 months (*P*<0.001, Figure 2A). Accordingly, 46% of the patients who had MPO-ANCA reappearance had a relapse, which was major in 33%. This frequency was higher when compared with the frequency of relapse and major relapse in patients who were persistently MPO-ANCA–positive (39% and 30%). On the other hand, patients who remained MPO-ANCA–negative did not relapse (Figure 2A). In our cohort, a persistently negative MPO-ANCA test result had a 100% negative predictive value for relapse, whereas MPO-ANCA reappearance or persistent positivity had positive predictive values for subsequent relapse of 64% and 45%, respectively.

The time to relapse according to the MPO-ANCA status stratified per remission-induction treatment is shown in Figure 2, B–D. There was no difference in the frequency of patients who turned persistently MPO-ANCA–negative when comparing the main remission-induction regimens (cyclophosphamide versus rituximab): 21/80=26% versus 7/43=16%, *P*=0.21 (Table 3).

### Discontinuation of Immunosuppression

Immunosuppression was maintained in 79 (50%) and withdrawn in 80 (50%) patients during follow-up. We compared the clinical characteristics and outcomes of patients in whom remission-maintenance therapy was withdrawn with those who maintained immunosuppression (Table 4). There were no significant differences in disease severity (assessed by Birmingham Vasculitis Activity Score/Wegener Granulomatosis) or organ involvement at baseline. Patients in whom immunosuppression was maintained were more frequently persistently MPO-ANCA–positive (52% versus 35%, *P*=0.03). Most of the patients in whom immunosuppression had been withdrawn had become MPO-ANCA–negative at some point during follow-up (65% versus 48%, *P*=0.03) (Table 4). In this cohort, there were no differences in the relapse rates and remaining outcomes whether maintenance immunosuppression was withdrawn.

**Table 4:** Clinical characteristics of patients with myeloperoxidase-ANCA–associated vasculitis with glomerulonephritis according to the status of immunosuppression (maintained immunosuppression versus withdrawn immunosuppression)

	Maintained Immunosuppression *n*=79 (49.7%)	Withdrawn Immunosuppression *n*=80 (50.3%)	*P* Value^a^
Age at diagnosis, median (IQR), yr	63 (56–72)	67 (55–73)	0.53
Male, *n* (%)	36 (46)	33 (41)	0.58
**Presentation, *n* (%)**			0.65
New diagnosis	64 (81)	67 (84)	
Relapse	15 (21)	12 (15)	
**AAV, *n* (%)**			0.18
MPA	74 (94)	70 (87)	
GPA	5 (6)	10 (13)	
BVAS/WG at diagnosis, median (IQR)	7 (7–9)	7 (7–10)	0.73
**Organ involvement classified using BVAS/WG at diagnosis, *n* (%)**			
Cutaneous	4 (5)	4 (5)	0.99
Mucous membranous/eye	3 (4)	5 (6)	0.48
Ear, nose, and throat	18 (23)	11 (14)	0.14
Cardiovascular	1 (1)	2 (2)	0.57
Gastrointestinal	0 (0)	0 (0)	—
Pulmonary	36 (46)	38 (47)	0.81
Kidney	79 (100)	80 (100)	—
Neurologic	3 (4)	1 (1)	0.52
**Remission-induction therapies, *n* (%)**			
Cyclophosphamide	46 (58)	34 (42)	0.05
Rituximab	15 (19)	28 (35)	0.02
MMF	15 (19)	12 (15)	0.50
**Remission-induction adjuvant therapies, *n* (%)**			
IV methylprednisolone at induction remission	43 (54)	47 (59)	0.58
Plasma exchange therapy	11 (14)	9 (11)	0.61
**Maintenance treatment, *n* (%)**			0.12
MMF	37 (49)	24 (31)	
Azathioprine	23 (30)	31 (40)	
Prednisone	8 (10)	16 (20)	
Rituximab	6 (8)	5 (7)	
Methotrexate	3 (4)	3 (4)	
Cyclophosphamide	0 (0)	2 (3)	
Time of remission-maintenance therapy, median (IQR)	22 (11–57)	31 (13–65)	0.43
**ANCA seronegative conversion after remission induction, *n* (%)**			
At remission	19 (24)	27 (34)	0.18
Total	38 (48)	52 (65)	0.03
Time to turn MPO-ANCA–negative (IQR), mo	6.3 (4.0–14.2)	5.3 (3.1–23.5)	0.04
**ANCA profile over time, *n* (%)**			0.03
Persistently negative	13 (17)	22 (28)	0.10
Reappearance	25 (32)	30 (38)	0.44
Persistently positive	41 (52)	28 (35)	0.03

IQR, interquartile range; AAV, ANCA-associated vasculitis; MPA, microscopic polyangiitis; GPA, granulomatosis with polyangiitis; BVAS/WG, Birmingham vasculitis activity score for Wegener granulomatosis; MMF, mycophenolate mofetil; IV, intravenous; MPO, myeloperoxidase.

Relapse occurred under remission-maintenance treatment in 35 (53%) patients or after remission maintenance had been withdrawn in 31 (47%) patients. Subtherapeutic doses of immunosuppressants were documented in 15 (42%) patients who relapsed under remission maintenance, nine patients were persistently positive and six had MPO-ANCA reappearance. The remission-maintenance regimens in those patients under subtherapeutic drugs were as follows: MMF ≤1000 mg/d in eight patients, azathioprine ≤100 mg/d in three patients, prednisone alone in three patients, and methotrexate ≤20 mg/wk in one patient. The average time to relapse after withdrawal of immunosuppression was 13 months. However, the median time from treatment withdrawal to relapse was shorter in patients who were persistently MPO-ANCA–positive compared with those who exhibited MPO-ANCA reappearance (0.80 versus 28.5 months, *P*=0.036).

Multivariable logistic regression showed that patients who received cyclophosphamide for remission induction and those who were persistently MPO-ANCA–positive were less likely to have remission-maintenance therapy withdrawn (OR, 0.44; 95% CI, 0.228 to 0.861; *P*=0.02 and OR, 0.42; 95% CI, 0.213 to 0.820; *P*=0.01, respectively). Using multivariable logistic regression, we also determined that, in patients who were withdrawn from remission-maintenance therapy, ENT involvement (OR, 6.10; 95% CI, 1.280 to 29.010; *P*=0.02) and the subsequent reappearance of MPO-ANCA (OR, 9.25; 95% CI, 3.126 to 27.361; *P*<0.01) were also independent predictive factors for relapse, just as for the entire cohort. In patients who stopped remission-maintenance treatment, the time from achieving remission to relapse was similar in patients who were persistently MPO-ANCA–positive compared with those who exhibited MPO-ANCA reappearance, whereas in patients who maintained remission-maintenance treatment, this time to relapse was shorter in patients who experienced MPO-ANCA reappearance (Figure 1B).

### Relapse Analysis According to Remission-Maintenance Treatment and MPO-ANCA Status

Patients who had turned and remained MPO-ANCA–negative were more likely to be withdrawn from immunosuppression maintenance without experiencing a relapse, with remission-induction treatment withdrawn in 63% (Table 5). The patients with MPO-ANCA reappearance during follow-up were at higher risk of relapse if remission maintenance was withdrawn (*P*<0.01): 64% relapsed, and in 61% of these, the relapse occurred after immunosuppression was withdrawn (Table 5). Similarly, 45% of patients with persistent MPO-ANCA positivity relapsed, and 65% of those were on remission-maintenance treatment at the time of the relapse (Table 5).

**Table 5:** Response to maintenance-remission therapy in patients with myeloperoxidase-ANCA–associated vasculitis with glomerulonephritis and kidney involvement according to myeloperoxidase-ANCA status

	MPO-ANCA Persistently Negative *n*=35 (22%)	MPO-ANCA Reappearance *n*=55 (35%)	MPO-ANCA Persistently Positive *n*=69 (43%)	*P* Value^a^
**Pattern of response to remission-maintenance therapy during all of the FU^b^^,^^c^**				<0.001
Stopped immunosuppression and did not relapse (median time of immunosuppression: 18.4 mo; median time of FU: 5.3 yr)	22 (63)	8 (15)	16 (23)	
Stopped immunosuppression and relapse (median time of immunosuppression; 22.7 mo; median time of FU: 6.4 yr)	0 (0)	20 (36)	11 (16)	
Maintained immunosuppression and did not relapse (median time of immunosuppression: 50.9 mo; median time of FU: 5.5 yr)	13 (37)	12 (22)	22 (32)	
Maintained immunosuppression and relapse (median time of immunosuppression: 51.5 mo; median time of FU: 8.9 yr)	0 (0)	15 (27)	20 (29)	
**MPO-ANCA status at the time of remission, *n* (%)**				<0.001
Positive	17 (49)	29 (53)	69 (100)	
Negative	18 (51)	26 (47)	—	
Time to turn MPO-ANCA–negative (IQR), mo	2.9 (1.9–3.9)	4.2 (2.6–5.5)	—	0.02
**Time of remission-maintenance treatment (IQR), mo**				
Total time of treatment	31.6 (15.4–91.0)	31.8 (14.7–65.5)	31.2 (12.2–102.1)	0.85
Time between stop remission-maintenance treatment and relapse	—	27.3 (2.27–41.6)	0.80 (0.0–19.1)	0.03
Time of FU (IQR), yr	6.5 (2.7–12.7)	10.1 (5.5–12.4)	4.9 (3.3–9.1)	<0.001

MPO, myeloperoxidase; FU, follow up; IQR, interquartile range.

a *P* value <0.05 is considered significant (Pearson χ^2^ test for categorical variables and Kruskal-Wallis test for continuous variables with skewed distribution).

b Median time of immunosuppression is statistically significantly different between categories (*P*<0.0001).

c Median time of FU after diagnosis of kidney disease is not statistically significantly different between categories (*P*=0.450).

### Decade Analysis

Treatment and outcome per decade were compared (Supplemental Table 3). Persistent MPO-ANCA negativity was more common in the first decade (50.0% versus 26.7%, *P*<0.01). This may be related to the more frequent use of cyclophosphamide for remission induction (70.4% versus 40%, *P*<0.01), which was also given for much longer periods than 4–6 months before 2003, when the Cyclophosphamide versus Azathioprine during Remission in ANCA-positive Systemic Vasculitis trial (CYCAZAREM) was published.

## Discussion

**Figure 3:**
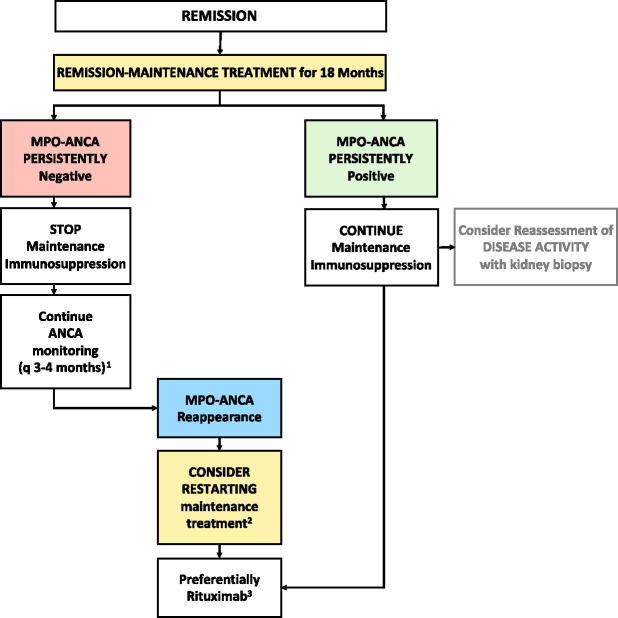
**Proposal for the clinical use of MPO-ANCA status for remission-maintenance guidance in MPO-ANCA–associated vasculitis with GN.** ^1^The suggested interval of serial MPO-ANCA determinations is based on the observed time to reappearance of MPO-ANCA after turning negative on treatment (median 5.5 [interquartile range 3.1–15.5] months). ^2^The time to start maintenance immunosuppression will depend on risk factors, such as age, baseline eGFR, previous relapses, and the higher risk of progression to kidney failure. ^3^In accordance with guidelines on the basis of randomized controlled trial results and supported by the relapses occurring on treatment with conventional disease-modifying drugs observed in our cohort, rituximab is recommended as the treatment of choice for remission maintenance.

Our study characterizes how MPO-ANCA status relates to the risk of relapse and how serial MPO-ANCA measurements can prove useful for remission-maintenance treatment decisions in patients with MPO-ANCA–associated vasculitis with GN. In our cohort, patients who turned and remained MPO-ANCA–negative did not relapse. By contrast, patients who remained MPO-ANCA–positive or exhibited MPO-ANCA reappearance after turning negative after remission-induction therapy had a higher risk for relapse. The discontinuation of remission-maintenance therapy did not translate into a higher number of relapses or kidney adverse events during follow-up, suggesting that remission-maintenance therapy can be stopped in patients who remained MPO-ANCA–negative, preventing unnecessary toxicity and adverse effects. Our results suggest that the institution of remission-maintenance therapy can be guided according to MPO-ANCA status and that MPO-ANCA serial measurements can help gauge the risk of relapse in patients who turned MPO-ANCA–negative. Hence, the threshold for restarting remission-maintenance treatment should be low in the context of MPO-ANCA reappearance (Figure 3).

ANCA-associated vasculitis is a relapsing-remitting disease.^23,24^ The risk of relapse has been correlated with suboptimal remission-maintenance treatment, such as using immunosuppressants in subtherapeutic doses or their premature discontinuation.^25^ Hence, the implicit need for ongoing appropriate and effective remission-maintenance treatment. We showed here that about 42% of the patients in whom relapses occurred during remission-maintenance treatment received doses that are considered subtherapeutic, and 60% of these were persistently MPO-ANCA–positive.^26^ By contrast, it is unclear if the length of remission-maintenance treatment has implications for minimizing relapse rates. The extension of remission maintenance with azathioprine beyond 18 months did not affect relapse free–survival in patients with PR3- and MPO-ANCA–associated vasculitis, and patients who stopped azathioprine before 18 months did not show a higher risk for relapse.^7,27^ Furthermore, the discontinuation of remission-maintenance therapy after a median period of treatment of 20 months did not show a higher risk of relapse.^28^ Therefore, the need and length of remission-maintenance treatment to mitigate relapse in patients who are MPO-ANCA–positive is unclear, and objective-guiding parameters are needed for the institution of an efficient and standardized approach.

Monitoring ANCA levels to predict disease activity or the occurrence of relapses in patients with ANCA-associated vasculitis has been controversial and remains a matter of debate.^29–37^ In 2012, a meta-analysis of the results of 18 studies addressing the effect of the rise in ANCA levels and of persistent ANCA for disease activity prediction in PR3- and MPO-ANCA–associated vasculitis concluded that serial ANCA measurements were not sufficiently robust to predict relapse, despite the correlation between rise and persistent ANCA positivity with relapse, with the inconsistency of time intervals of ANCA measurements between studies being one of the major limitations.^18^ In patients with ANCA-associated vasculitis with GN, Kemna *et al.*^15^ concluded that it is the proportion of patients with kidney involvement in a specific cohort of patients with ANCA-associated vasculitis that will determine whether ANCA measurements are of value for relapse prediction during follow-up. Subsequently, a subanalysis of the Rituximab versus Cyclophosphamide (RAVE) trial showed that serial determinations of PR3-ANCA in patients with complete remission were informative of the risk of severe relapse, particularly in patients with disease manifestations of capillaritis, including GN.^20^ Importantly, meaningful analyses of MPO-ANCA patients could not be performed because the number of events was too low among the MPO-ANCA–positive patients during the trial period.^20^ In contrast to the studies included in the meta-analysis, in which the focus was on titer increases, ANCA reappearance after prior negative status has been associated with a higher risk of relapse, and it has been suggested as a valuable biomarker for relapse prediction.^15,19,20,37–40^ MPO-ANCA reappearance was associated with an OR of 23.2 for the occurrence of relapse in a nationwide cohort of 271 Japanese patients with microscopic with polyangiitis, granulomatosis with polyangiitis (GPA), and eosinophilic GPA, in which 221 patients had kidney involvement.^39^ In addition, patients with a persistent ANCA-negative status after remission-induction treatment are reported to be at a lower risk of relapse.^19,38,39^ Finally, it was observed that the timing at which ANCA negativity was achieved could affect relapse risk prediction.^41^ Morgan MD *et al.* conducted a study including patients who received cyclophosphamide for remission-induction treatment and were switched for remission-maintenance therapy after achieving remission in the Daily Oral versus pulse Cyclophosphamide as Therapy for ANCA-associated Systemic Vasculitis (CYCLOPS) and the International Mycophenolate Mofetil Protocol to Reduce Outbreaks of Vasculitides (IMPROVE) trials. The authors showed that achievement of ANCA negativity at the time of remission was associated with a reduced risk of relapse.^41–43^ ANCA status appears to correlate with relapse risk, particularly in patients with MPO-ANCA–associated vasculitis with GN.

The Maintenance of Remission using Rituximab in Systemic ANCA-associated Vasculitis trial (MAINRITSAN) showed that patients treated with cyclophosphamide for remission induction who received azathioprine (2 mg/kg per day for 12 months and then 1.5 mg/kg per day for 6 months and 1 mg/kg per day for 4 months) for remission maintenance had a six times higher risk of relapse compared with patients who received rituximab (500 mg IV×2 followed every 6 months by 500 mg IV at 6, 12, and 18 months after the first infusion).^44^ In addition, the Rituximab Vasculitis Maintenance Study trial showed that in relapsing patients who received rituximab (RITAZAREM) for remission induction, its use for remission maintenance (1000 mg IV every 4 months until 24 months) was associated with a lower relapse rate compared with patients treated with azathioprine (HR, 0.36; 95% CI, 0.23 to 0.57; *P*<0.001). No differences in adverse events between these treatment alternatives were found in either trial.^44,45^ Consequently, rituximab is now the preferred remission-maintenance strategy.^8^

Our study corroborates and expands on the prior observations suggesting that serial ANCA determinations are useful to gauge the relapse risk of patients with MPO-ANCA–associated vasculitis with GN. On the basis of our results reported here and the data about MPO-ANCA and relapse risk derived from other studies, paired with what we now know about variable efficacy of rituximab versus azathioprine (and by proxy, MMF and methotrexate), we propose an algorithm that utilizes the serial determination of the MPO-ANCA status after remission induction to guide the institution of effective remission-maintenance treatment in patients with MPO-ANCA–associated vasculitis with GN (Figure 3). This suggested clinical management approach is on the basis of three important findings: (*1*) patients who turn and remain MPO-ANCA–negative after an average of 18 months of remission-induction treatment do not relapse—therefore the decision to keep the remission-maintenance therapy can be delayed until MPO-ANCA reappearance is detected by serial MPO-ANCA monitoring; (*2*) in those patients who exhibit MPO-ANCA reappearance, remission-maintenance treatment with rituximab should be instituted and maintained until MPO-ANCA turns negative again, particularly in patients at a higher risk of progression to kidney failure; and (*3*) finally, effective remission maintenance should be initiated in all the patients who remain MPO-ANCA–positive after remission-induction treatment. Discontinuation of remission-maintenance therapy in patients who have remained in long-term remission despite persistent MPO-ANCA positivity should be carefully pondered, as about 40% of these patients will relapse. Finally, the use of MPO-ANCA status, as opposed to MPO-ANCA titer changes, to guide clinical decisions seems advantageous because it is independent of different ANCA testing methodologies and thus allows standardized decisions that can be replicated across different centers, leading to homogeneous practices in remission-maintenance treatment in ANCA-associated vasculitis with GN.

This study has limitations inherent to its retrospective design. First, our cohort consists of a Midwestern US White population with predominantly Scandinavian and Northern European ethnic backgrounds; therefore, the results may not be generalizable. Second, the standard of care between 1996 and 2003 was to use cyclophosphamide for maintenance remission, with azathioprine use becoming widespread only after the publication of the Cyclophosphamide versus Azathioprine during Remission in ANCA-positive Systemic Vasculitis trial in 2003,^46^ and with rituximab more widely accepted (CYCAZAREM) after the publication of the Maintenance of Remission using Rituximab in Systemic ANCA-associated Vasculitis trial in 2014.^44^ However, the use of MMF as remission-maintenance strategy (MAINRITSAN) is an option in patients with MPO-ANCA–associated vasculitis with GN, in particular in the ones with mild to moderate disease or intolerant to azathioprine.^47^ Moreover, remission-maintenance treatment choices were made according to the best medical judgment and did not follow a preestablished protocol. In addition, in patients with persistently positive MPO-ANCA and suggestion of kidney disease activity (*i.e.*, reduced kidney function, persistent hematuria) despite adequate immunosuppression, repeat kidney biopsies to rule out ongoing vasculitis was not performed because this is not our clinical practice. However, we acknowledge that this can be considered. Improvements in ANCA detection methodologies or clinical care over time could not be quantified in our study. Therefore, our study reflects the reality of routine clinical practice and underscores the need for a more standardized approach. The proposed algorithm might contribute to a more homogeneous pattern of clinical decisions.

Despite these limitations, this is the largest cohort of patients with MPO-ANCA–associated vasculitis and active kidney involvement with long follow-up, allowing a detailed analysis of clinical characteristics and outcomes in response to different remission-induction and remission-maintenance treatments in real clinical practice, whereas another important study was on the basis of national registry.^39^ Although the treatment of our patients did not follow a strictly standardized protocol, they were managed by a group of experts in ANCA-associated vasculitis who followed consistent and timely practice patterns for the management of ANCA-associated vasculitis with GN. These results are applicable to patients with MPO-ANCA–associated vasculitis and kidney involvement, a considerable portion of the patients with ANCA-associated vasculitis.^48,49^

In conclusion, our results indicate that patients who turn and remain MPO-ANCA–negative after remission-induction treatment are not at risk for relapse despite discontinuation of remission-maintenance therapy for as long as they remain MPO-ANCA–negative. Once MPO-ANCA reappearance occurs, these patients are at risk for relapse at a similar rate to those patients who remain MPO-ANCA–positive. Our results suggest that continued serial MPO-ANCA monitoring in patients with MPO-ANCA–associated vasculitis with GN can be used to guide remission-maintenance treatment. The most effective remission-maintenance therapy should be chosen for these patients. Our observations require confirmation in prospective studies.

## Supplementary Material

## Data Availability

All data used in this study are available in this article.

## Supplemental Material

Supplemental Material and Methods.

Supplemental Figure 1. STROBE (STrengthening the Reporting of OBservational studies in Epidemiology) flow chart for the selection of the patient with active and severe renal involvement in ANCA-associated vasculitis (AAV).

Supplemental Figure 2. Kaplan–Meier plots of relapse over 60 months according to the remission-maintenance treatment strategy.

Supplemental Figure 3. Kaplan–Meier plots of relapse according to remission-induction treatment.

Supplemental Table 1. Demographic and clinical characteristics of patients with MPO-AAV-GN (*n*=159).

Supplemental Table 2. Outcomes of patients with MPO-AAV-GN (*n*=159).

Supplemental Table 3. Comparison of treatment and outcomes of patients according to the period of time of AAV-GN diagnosis.
