# Supplementary material for: Maintenance of Remission and Risk of Relapse in Myeloperoxidase-Positive ANCA-Associated Vasculitis with Kidney Involvement
Source: Clin J Am Soc Nephrol. 2022 Jan 18;18(1):47–59. doi: 10.2215/CJN.06460622 (PMC10101626; doi:10.2215/CJN.06460622)
Supplement: Supplementary file 1 [file cjasn-18-047-s001.pdf]

Supplemental Material

Supplemental Material and Methods

Supplemental Figure 1. STROBE (STrengthening the Reporting of OBservational studies in Epidemiology) flowchart for the selection of the patient with active and severe renal involvement in Anti-Neutrophil Cytoplasmic Antibodies (ANCA) associated vasculitis (AAV).

Supplemental Figure 2. Kaplan Meier plots of relapse over 60 months according to the remission-maintenance treatment strategy.

Supplemental Figure 3. Kaplan Meier plots of relapse according with remission induction treatment.

Supplemental Table 1. Demographic and clinical characteristics of patients with MPO-AAV-GN (n=159).

Supplemental Table 2. Outcomes of patients with MPO-AAV-GN (n=159).

Supplemental Table 3. Comparison of treatment and outcomes of patients according with the period of time of AAV-GN diagnosis.

Supplemental Material

Supplemental Material and Methods

Study design. A single-center retrospective cohort study of all consecutive patients with AAV-associated active renal disease (AAV-GN) evaluated at Mayo Clinic from January 1, 1996, to December 31, 2015.[1] The "Mayo Clinic ANCA-associated vasculitis cohort" comprised a total of 1830 patients with a diagnosis of AAV managed by vasculitis experts. Patients with renal involvement were identified by applying an internal search engine (Advanced Cohort Explorer - ACE) using the following keywords: "kidney disease," "renal failure," "pauci-immune glomerulonephritis," "hematuria," "proteinuria," and "renal biopsy." The study was approved by the Mayo Clinic institutional review board (#14-0056411).

Patient characteristics. All data were abstracted retrospectively from electronic medical records and included demographic characteristics, comorbidities, laboratory findings, biopsy results, therapies, and outcomes. The date of kidney involvement diagnosis (reference date) was registered to calculate outcomes time-points. The Birmingham Vasculitis Activity Score for Wegener´s Granulomatosis (BVAS/WG) was used to quantify disease severity at presentation and during follow-up.[2]

MPO-ANCA positive patients with newly diagnosed AAV or relapsing disease with active kidney involvement and fulfilling the American College of Rheumatology (ACR) criteria and Chapel Hill consensus definition for granulomatosis with polyangiitis (GPA) and microscopic polyangiitis (MPA) were included (Fig.1).[3,4] The clinicopathologic diagnosis originally assigned by the clinician was accepted. A known remission status, a defined remission-maintenance strategy and a minimum of 6 months of follow up after achieving remission was required for inclusion.

1. Casal Moura M et al. Efficacy of Rituximab and Plasma Exchange in Antineutrophil Cytoplasmic Antibody-Associated Vasculitis with Severe Renal Disease. *J Am Soc Nephrol.* 2020;
2. Stone JH et al. A disease-specific activity index for Wegener's granulomatosis: modification of the Birmingham Vasculitis Activity Score. International Network for the Study of the Systemic Vasculitides (INSSYS). *Arthritis Rheum.* 2001;
3. Leavitt RY et al. The American College of Rheumatology 1990 criteria for the classification of Wegener granulomatosis. *Arthritis Rheumatol.* 1990;
4. Jennette JC et al. 2012 revised International Chapel Hill Consensus Conference Nomenclature of Vasculitides. *Arthritis Rheum.* 2013.

Outcome assessment. Remission was defined by a BVAS/WG of 0 independent of the prednisone dose, and sustained remission was defined by a BVAS/WG of 0 present for more than 6 months independently of the prednisone dose. These events were assessed at 6 months and during follow-up. All patients included in the analysis achieved remission. Relapse was defined by an increase of BVAS/WG > 1 after remission had been achieved, that resulted in therapy changes (increases in doses of remission-maintenance therapy or the start of a new remission-induction cycle). Time to relapse after remission (independently of ANCA-status at the time of remission); the number of relapses after the achievement of remission; type of relapse (major [BVAS/WG>3] or minor); the organ involvement (renal versus non-renal); and the BVAS/WG at the time of relapse were recorded. Only the first incident event of relapse (index relapse) was included in the analyses. Follow-up time was recorded from diagnosis of kidney involvement until last evaluation at Mayo Clinic. No significant losses of follow-up was observed. Kidney failure was defined as eGFR <15 mL/min/1.73m^2 or the need to initiate kidney replacement therapy.[5]

Remission-maintenance therapies. The remission-maintenance treatment used was not defined by a pre-established protocol but decided by the treating physicians according to the best clinical judgment.[6-14] Glucocorticoid sparing immunosuppression maintenance therapy regimens used were: (i) mycophenolate mofetil, oral, dose range from 1000 to 2000 mg/day (therapeutic dose > 1000 mg/day); (ii) azathioprine, oral, 2 mg/kg/day (therapeutic dose >100 mg/day); (iii)

5. Levey AS et al. Nomenclature for kidney function and disease: report of a Kidney Disease: Improving Global Outcomes (KDIGO) Consensus Conference. *Kidney Int.* 2020;
6. Kitching R et al. ANCA-associated vasculitis. Nat Rev Dis Primers. 2020;
7. Jayne D et al. A Randomized Trial of Maintenance Therapy for Vasculitis Associated with Antineutrophil Cytoplasmic Autoantibodies. *N Engl J Med.* 2003;
8. Pagnoux C et al. Azathioprine or Methotrexate Maintenance for ANCA-Associated Vasculitis. *N Engl J Med.* 2008.
9. Puechal X et al. Long-Term Outcomes Among Participants in the WEGENT Trial of Remission-Maintenance Therapy for Granulomatosis With Polyangiitis (Wegener's) or Microscopic Polyangiitis. *Arthritis Rheumatol.* 2016.
10. Hiemstra TF et al. Mycophenolate Mofetil vs Azathioprine for Remission Maintenance in Antineutrophil Cytoplasmic Antibody–Associated Vasculitis. A Randomized Controlled Trial. *JAMA.* 2010;
11. Guillevin L. Rituximab versus azathioprine for maintenance in ANCA-associated vasculitis. *N Engl J Med.* 2014;
12. Terrier B et al. Long-term efficacy of remission-maintenance regimens for ANCA-associated vasculitides. *Annals Rheum Dis.* 2018.
13. Charles P et al. Comparison of individually tailored versus fixed-schedule rituximab regimen to maintain ANCA-associated vasculitis remission: results of a multicentre, randomised controlled, phase III trial (MAINRITSAN2). *Ann Rheum Dis.* 2018.
14. Smith RM. Rituximab as therapy to induce remission after relapse in ANCA-associated vasculitis. *Ann Rheum Dis.* 2020.

methotrexate, subcutaneous, 0.3 mg/kg/week progressively increased to 25 mg/week (therapeutic dose >20 mg/week); (iv) cyclophosphamide, oral, 1.5 mg/Kg/day; (v) rituximab, intravenous, 500 or 1000 mg two weeks apart, scheduled at fixed intervals or tailored to B cell return (considered immunosuppressed if B cell counts = 0) and ANCA serology.[6-14] Low dose prednisone was used variably as a remission maintenance agent in this cohort.

[5.]Levey AS et al. Nomenclature for kidney function and disease: report of a Kidney Disease: Improving Global Outcomes (KDIGO) Consensus Conference. *Kidney Int.* 2020;

[6.]Kitching R et al. ANCA-associated vasculitis. Nat Rev Dis Primers. 2020;

[7.]Jayne D et al. A Randomized Trial of Maintenance Therapy for Vasculitis Associated with Antineutrophil Cytoplasmic Autoantibodies. *N Engl J Med.* 2003;

[8.]Pagnoux C et al. Azathioprine or Methotrexate Maintenance for ANCA-Associated Vasculitis. *N Engl J Med.* 2008.

[9.]Puechal X et al. Long-Term Outcomes Among Participants in the WEGENT Trial of Remission-Maintenance Therapy for Granulomatosis With Polyangiitis (Wegener's) or Microscopic Polyangiitis. *Arthritis Rheumatol.* 2016.

[10.]Hiemstra TF et al. Mycophenolate Mofetil vs Azathioprine for Remission Maintenance in Antineutrophil Cytoplasmic Antibody–Associated Vasculitis. A Randomized Controlled Trial. *JAMA.* 2010;

[11.]Guillevin L. Rituximab versus azathioprine for maintenance in ANCA-associated vasculitis. *N Engl J Med.* 2014;

[12.]Terrier B et al. Long-term efficacy of remission-maintenance regimens for ANCA-associated vasculitides. *Annals Rheum Dis.* 2018.

[13.] Charles P et al. Comparison of individually tailored versus fixed-schedule rituximab regimen to maintain ANCA-associated vasculitis remission: results of a multicentre, randomised controlled, phase III trial (MAINRITSAN2). *Ann Rheum Dis.* 2018.

[14.]Smith RM. Rituximab as therapy to induce remission after relapse in ANCA-associated vasculitis. *Ann Rheum Dis.* 2020.

MPO-ANCA status classification and serial determinations. Patients were longitudinally analyzed and classified into 3 categories according to their MPO-ANCA status at the end of the follow-up: (i) sustained seronegative: patients that turned MPO-ANCA negative at any time during follow-up and remained negative; (ii) reappearance: patients that turned MPO-ANCA positive after becoming MPO-ANCA negative; and (iii) persistently positive, patients who remained MPO-ANCA positive throughout the entire follow-up period. The MPO-ANCA status at the time of remission (BVAS/WG = 0) was also recorded. MPO-ANCA serial determinations at all available time points were recorded. ANCA specificity was determined by solid-phase immunoassays and complemented by indirect immunofluorescence characterization of a perinuclear pattern (p-ANCA).[15]

Serial MPO-ANCA determinations were recorded at all available time points. MPO-ANCA-specificity was determined by solid phase immunoassays and complemented by indirect immunofluorescence (IIF) characterization of a perinuclear pattern (p-ANCA). Over time, 4 assays were used to determine MPO-ANCA, and we analyzed each patient’s results and status along time with the assay used in mind:

a) On 10/16/1991, the lab implemented the first anti-MPO assay (BioCarb Diagnostics distributed by Scimedx Corp). For this assay, <10 U is normal.

b) On 2/20/2003, the lab changed methods to Scanlisa MPO Antibody assay from SciMedx Corp. The cut-offs for this assay are: Negative </= 5.0 U/mL, Equivocal 5.1-14.9 U/mL, and Positive >/= 15.0 U/mL.

c) On 9/19/2006, the lab changed methods again to Varelisa MPO kit (Pharmacia Diagnostics. The cut-offs for this assay are: Negative < 6.0 U/mL, Equivocal 6.0-9.0 U/mL, and Positive > 9.0 U/mL.

15. Russell KA et al. Detection of anti-neutrophil cytoplasmic antibodies under actual clinical testing conditions. *Clin Immunol*. 2002

d) On 11/10/2008, the lab changed to our current method (BioPlex by BioRad). The cut-offs for the current assay are: Negative < 0.4 U, Equivocal 0.4-0.9 U, and Positive >/= 1.0 U/mL

When borderline or equivocal by MPO-specific immunoassay, the status was determined by combining the MPO-ANCA serology with IIF results: if both positive – positive; if equivocal result by MPO-specific immunoassay and IIF negative – negative; if equivocal by MPO-specific immunoassay and positive on IIF– positive.

Immunosuppression withdrawal and relation with relapse. We classified the patients into 4 groups: (i) patients who were withdrawn from therapy and did not relapse; (ii) patients who were withdrawn from therapy and relapsed; (iii) patients who were not withdrawn from therapy and did not relapse; (iv) patients who were not withdrawn from therapy and relapsed.

Statistical analysis. Categorical variables were presented as count (percent), while continuous variables were presented as mean (standard deviation) if they were normally distributed as determined by the Shapiro-Wilk test or as median (interquartile range [IQR]) if not normally distributed. Pearson's chi-square test was used to compare categorical variables between groups. The Bonferroni correction for multiple comparisons was applied when more than two categories of categorical variables were compared. For comparison of continuous variables between two groups, an unpaired Student's t-test for independent samples was used for distributions consistent with normality, and the Mann-Whitney U test was used otherwise; for comparison of continuous variables between more than two groups, an ANOVA test was used for distributions consistent with normality, and the Kruskal-Wallis test was used otherwise. There was no imputation or removal of data due to missing data since the variables of interest were complete for all the patients.

Logistic regression models were developed to examine the predictive role of the baseline clinical organ involvement and remission-induction treatment impact on therapy withdrawal. Variables were considered for the multivariable logistic regression models if they occurred before the development of the outcome of interest, had <10% of missing values, had p values < 0.05 in the univariate analysis, and were clinically plausible. The final model was determined using both clinical and statistical criteria, considering collinearity, interaction, and the number of patients who experienced the outcome of interest. The odds ratios (OR) with a 95% confidence interval (95% CI) were reported when appropriate.

The Kaplan Meier method was used to assess the cumulative incidence of relapse after achieving remission. Cox proportional hazards regression models were used to determine predictive factors for relapse at 60 months. We report the HR with a 95% CI when appropriate.[16] The final model was determined using both clinical and statistical criteria, considering collinearity, interaction, and the number of patients who experienced the outcome of interest. We evaluated the following covariates: age at diagnosis, gender, new diagnosis vs. previous relapse, type of AAV (MPA vs. GPA), BVAS/WG score, type of organ involvement on the BVAS/WG score, alveolar hemorrhage, arterial hypertension, diabetes mellitus, dyslipidemia, BMI > 30 kg/m2, hemoglobin, eGFR at diagnosis, eGFR at diagnosis < 30 mL/min/1.73m2, eGFR at diagnosis < 15 mL/min/1.73m2, remission-induction therapies, remission-induction adjuvant therapies, type of remission-maintenance therapies, ANCA status. and keeping or stopping immunosuppression. The final model included only variables that were statistically significant in the univariable analysis (p<0.05) or that are known to be associated with higher risk of relapse (for instance, ear nose and throat involvement). We performed backward method for selection of the model. The frequency of death prior to 60 months was ≤10%, and therefore, the risk of being a competitive event over such a long period of time was considered negligible.

16. Hernan MA: The hazards of hazard ratios. *Epidemiology*. 2010

IBM® SPSS® Statistics for MacOS, version 26 (IBM, Armonk, NY, USA) was used for all data analysis.

Supplemental Figures

Supplemental Figure 1

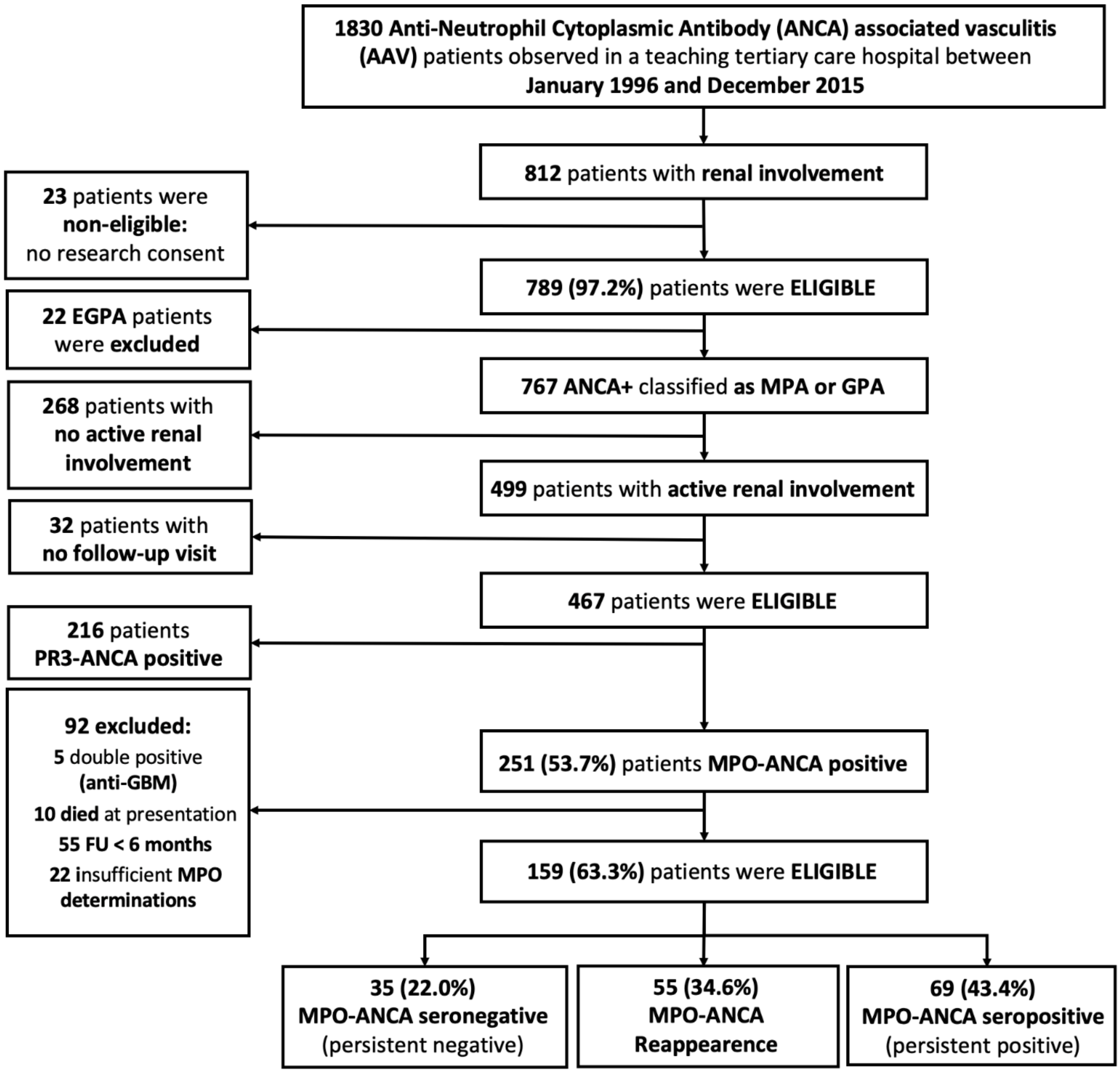

Supplemental Figure 2

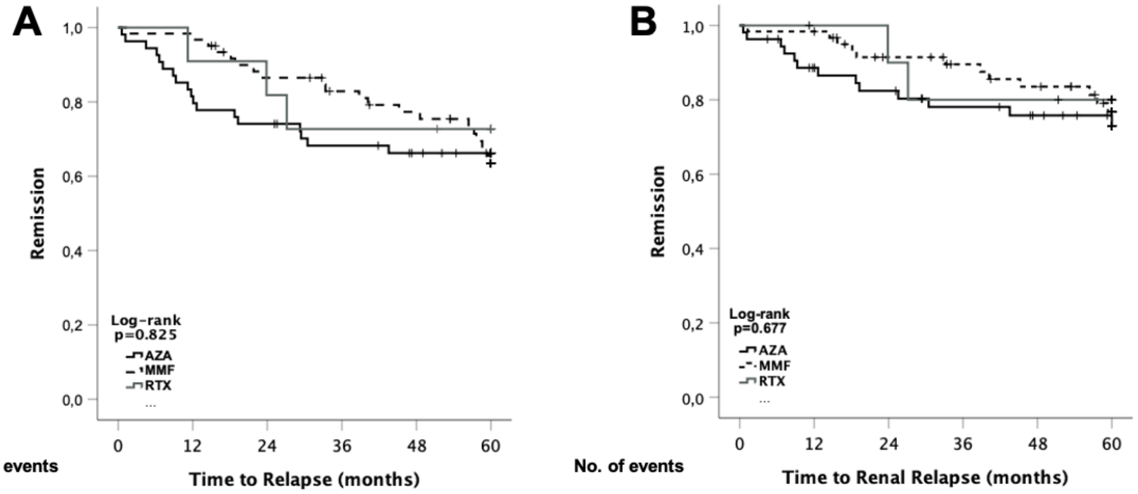

No. of events	0	12	24	36	48	60
AZA (n = 54)	0	10	14	17	18	18
MMF (n = 61)	0	2	8	10	13	20
RTX (n = 11)	0	1	2	3	3	3

No. of events	0	12	24	36	48	60
AZA (n = 54)	0	6	9	11	12	13
MMF (n = 61)	0	1	5	6	9	12
RTX (n = 11)	0	0	1	2	2	2

Supplemental Figure 3

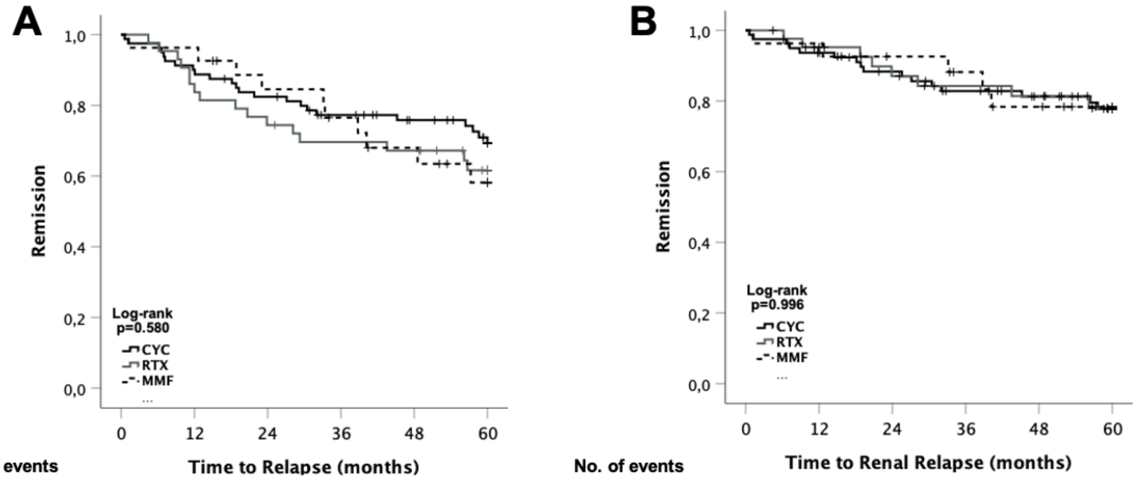

No. of events	0	12	24	36	48	60
CYC (n = 80)	0	8	15	23	30	38
RTX (n = 43)	0	6	11	14	15	23
MMF (n = 27)	0	1	6	9	12	16

No. of events	0	12	24	36	48	60
CYC (n = 80)	0	5	9	13	14	16
RTX (n = 43)	0	2	5	6	7	8
MMF (n = 27)	0	1	2	3	5	5

Supplemental Figures Legends

Supplemental Fig. 1 - STROBE (STrengthening the Reporting of OBservational studies in Epidemiology) flowchart for the selection of the patient with active and severe renal involvement in Anti-Neutrophil Cytoplasmic Antibodies (ANCA) associated vasculitis (AAV). Active renal involvement was defined by the presence of either (1) active, biopsy-proven, pauci-immune glomerulonephritis; (2) red blood cell casts on urine microscopy, or (3) rise in serum creatinine (SCr) > 30% (or >25% decline in creatinine clearance) attributed to active vasculitis. Patients were grouped according to the status of MPO-ANCA. (Abbreviations used: EGPA - eosinophilic granulomatosis with polyangiitis, FU - follow-up, GBM – glomerular basal membrane, GPA - granulomatosis with polyangiitis, MPA - microscopic polyangiitis).

Supplemental Fig. 2 - Kaplan Meier plots of relapse over 60 months according to the remission-maintenance treatment strategy. 1A: All types of relapse per remission-maintenance treatment (AZA vs. MMF vs. RTX) – 18 vs. 20 vs. 3 events, mean time to event – 44 vs. 51 vs. 49 months, p = 0.825; 1B: Renal relapse per remission-maintenance treatment (AZA vs. MMF vs. RTX) - 13 vs. 12 vs. 2 events, mean time to event – 49 vs. 54 vs. 53 months, p = 0.677 (Abbreviations: AZA – azathioprine; MMF – mycophenolate mofetil; RTX – rituximab).

Supplemental Fig. 3 - Kaplan Meier plots of relapse according with remission induction treatment. 2A: Any relapse. 2B: Renal relapse. (CYC, cyclophosphamide; RTX, rituximab, MMF, mycophenolate mofetil).

Supplemental Tables

Supplemental Table 1 - Demographic and clinical characteristics of patients with MPO-AAV-GN (n=159).

	MPO-ANCA positive<br>n = 159
Age at renal involvement diagnosis, median (IQR) years	65 (56-73)
Male, n (%)	69 (43)
Disease presentation, n (%)	
AAV new diagnosis	131 (82)
AAV relapse	28 (18)
AAV, n (%)	
MPA	144 (91)
GPA	15 (9)
BVAS/WG at renal involvement diagnosis, median (IQR)	7 (7-9)
Organ involvement classified using BVAS/WG at diagnosis, n (%)	
Cutaneous	8 (5)
Mucous membranous/eye	8 (5)
Ear, nose and throat	29 (18)
Cardiovascular	3 (2)
Gastrointestinal	0 (0)
Pulmonary	74 (47)
Renal	159 (100)
Neurologic	4 (3)
Alveolar hemorrhage BVAS/WG at diagnosis, n (%)	30 (19)
Cardiovascular risk factors, n (%)	
Arterial hypertension	111 (70)
Diabetes mellitus	21 (13)
Dyslipidemia	58 (37)
BMI > 30 Kg/m^2	56 (35)
Laboratory findings	
Hemoglobin, mean (sd) g/dL	10.4 (1.9)
SCr at diagnosis, median (IQR) mg/dL	2.1 (1.5-3.5)
eGFR at diagnosis, median (IQR), mL/min/1.73m^2	25.4 (15.7-43.3)
eGFR at diagnosis < 30 mL/min/1.73m^2, n (%)	92 (58)
eGFR at diagnosis < 15 mL/min/1.73m^2, n (%)	37 (23)
Dialysis, n (%)	19 (12)
Biopsy proven, n (%)	141 (89)
Intervention	
Remission-induction treatment, n (%)	
Cyclophosphamide	80 (50)
Rituximab	43 (27)
Mycophenolate mofetil	27 (17)
Prednisone	7 (4)
Methotrexate	2 (1)
Corticosteroids, n (%)	
IV methylprednisolone	90 (51)
Oral prednisone	69 (43)
Plasma exchange therapy, n (%)	20 (13)
Maintenance treatment, n (%)	
Mycophenolate mofetil	61 (39)
Azathioprine	54 (34)
Prednisone	25 (16)
Rituximab	11 (7)
Methotrexate	6 (4)

Cyclophosphamide	2 (1)
Time of maintenance treatment, months (IQR)	
Total time of treatment	31.2 (12.8-88.2)
ANCA seronegative conversion after remission-induction, n (%)	
At remission	46 (29)
Total	90 (57)
Time to seronegative conversion, months (IQR)	3.0 (0.5 – 9.6)
ANCA profile overtime, n (%)	
Persistently negative	35 (22)
Reappearance	55 (35)
Persistently positive	69 (43)

Abbreviations: AAV - antineutrophil cytoplasmic antibody associated vasculitis; ANCA - anti-neutrophil cytoplasmic antibody; BVAS/WG - Birmingham vasculitis activity score for Wegener granulomatosis; eGFR – estimated glomerular filtration rate; ESR - erythocyte sedimentation rate; GPA - ganulomatosis with polyangiitis; IQR - interquartile range; IV – intravenous; MPA - microscopic polyangiitis; MPO - myeloperoxidase; n- number; PLEX – Plasma exchange; PR3 - proteinase 3; SCr - serum creatinine.

Supplemental Table 2 - Outcomes of patients with MPO-AAV-GN (n=159).

	MPO-ANCA positive<br>n = 159
**Vasculitis, n (%)**	
Remission	
6 months	130 (82)
Total	159 (100)
Time to remission, median (IQR) months	4.1 (2.7 – 5.9)
Relapse	
60 months	52 (33)
Total	66 (42)
Time to relapse, median (IQR) month	38.9 (12.1 – 58.8)
Renal relapse	42 (27)
Major relapse	57 (36)
Death	
Total	44 (28)
Time to death, median (IQR) months	104 (66.4 – 152.6)
**Renal, n (%)**	
Kidney failure	
Total	24 (15)
Dialysis	19 (79)
Recovery of renal function after dialysis, n (%)	1 (0.6)
**Combined events of kidney failure and/or death, n (%)**	
Total	48 (30)
Time to Combined Events, median (IQR) months	41.6 (5.4 – 70.0)
Time of FU after renal involvement, median (IQR) years	6.1 (3.8 – 11.3)
Time of FU, median (IQR) years	7.7 (4.4 – 12.1)

Abbreviations: AAV – anti-neutrophil cytoplasmic antibody associated vasculitis; eGFR – estimated glomerular filtration rate; ESRD - end-stage renal disease; FU – follow-up; IQR – interquartile range; n - number.

Supplemental Table 3 – Comparison of treatment and outcomes of patients according with the period of time of AAV-GN diagnosis.

	1996-2005<br>n = 54 (34%)	2006-2015<br>n = 105 (68%)	**p-value*
ANCA profile overtime, n (%)			<0.01
Persistently negative	27 (50.0)	28 (26.7)	
Reappearance	18 (33.3)	17 (16,2)	
Persistently positive	9 (16.7)	60 (57.2	
Remission-induction therapies, n (%)			
Cyclophosphamide	38 (70.4)	42 (40.0)	<0.01
Rituximab	3 (5.6)	41 (39.0)	<0.01
Mycophenolate mofetil	7 (13.0)	20 (19.0)	0.33
Remission-induction adjuvant therapies, n (%)			
IV methylprednisolone at induction remission	19 (35.2)	71 (67.6)	<0.01
Plasma exchange therapy	2 (3.7)	18 (17.1)	0.02
Maintenance treatment, n (%)			0.12
Mycophenolate mofetil	21 (38.9)	40 (38.1)	
Azathioprine	20 (37.0)	34 (32.4)	
Prednisone	9 (16.7)	16 (15.2)	
Rituximab	0 (0.0)	11 (10.5)	
Methotrexate	2 (3.7)	4 (3.8)	
Cyclophosphamide	2 (3.7)	0 (0.0)	
Time of remission-maintenance therapy, median (IQR)	35 (14-144)	26 (12-64)	0.06
Outcomes			
Remission			
Relapse	42 (79)	90 (88)	0.14
Total	23 (42.6)	41 (41.0)	0.84
60 months	19 (35.2)	33 (31.4)	0.63
Kidney failure	9 (17)	15 (14)	0.69
Death	22 (54)	22 (21)	0.01

Abbreviations: IQR - interquartile range; IV – intravenous; n- number.
